# Seasonal variations in cause-specific mortality and transition to renal replacement therapy among patients with end-stage renal disease

**DOI:** 10.1038/s41598-020-59153-6

**Published:** 2020-02-11

**Authors:** Shunsuke Goto, Takayuki Hamano, Satoshi Ogata, Ikuto Masakane

**Affiliations:** 1 Committee of Renal Data Registry, Japanese Society for Dialysis Therapy, Tokyo, Japan; 2 Division of Nephrology and Kidney Center, Kobe University Graduate School of Medicine, Kobe, Japan; 3 Department of Nephrology, Nagoya City University Graduate School of Medical Sciences, Nagoya, Japan; 4 Department of Clinical Nutrition, Hiroshima International University, Kure, Japan; 5 Department of Nephrology, Honcho Yabuki Clinic, Yamagata, Japan

**Keywords:** Haemodialysis, Peritoneal dialysis

## Abstract

Despite some studies showing seasonal variations in mortality and the transition to renal replacement therapy in patients with end-stage renal disease, detailed evidence is still scarce. We investigated seasonal variations in patients with end-stage renal disease using a large Japanese database for dialysis patients. We compared the fractions of all-cause and cause-specific mortality and the transition to renal replacement therapy among seasons and performed a mixed-effects Poisson regression analysis to compare the mortality among seasons after adjustment for some variables. The initiation of hemodialysis was highest in winter and lowest in summer. Seasonality in the initiation of peritoneal dialysis and transition to kidney transplantation differed from hemodialysis. All-cause mortality was highest in the winter and lowest in the summer. Death from coronary artery disease, heart failure, cerebral hemorrhage, and infectious pneumonia had similar seasonality, but death from cerebral infarction, septicemia, or malignant tumor did not have similar seasonality. In conclusion, the initiation of hemodialysis, all-cause mortality, and mortality from coronary heart disease, heart failure, cerebral hemorrhage, and infectious pneumonia were significantly highest in winter and lowest in summer. However, the initiation of peritoneal dialysis, transition to kidney transplantation, or mortality from cerebral infarction, septicemia, or malignant tumor did not have similar seasonal variations.

## Introduction

Seasonal variations of various diseases in the general population have been investigated in numerous papers^1–6^. For example, heart failure, acute myocardial infarction, and sepsis owing to respiratory infection have a peak in the winter, whereas mortality from cancer has little or no seasonal variation. However, the seasonal variation in the mortality from each disease in patients with end-stage renal disease (ESRD) remains unclear, although some studies have investigated the seasonal variations in all-cause mortality or mortality from cardiovascular disease (CVD), infectious disease, or cancer^7–10^. In addition, because the mortality rate in patients with ESRD is substantially higher and the distribution of cause of death differs^11^, the results in the general population should not be extrapolated to ESRD.

There is also a lack in studies investigating the seasonal variation in the transition to renal replacement therapy (RRT)^7,12,13^. Two Japanese studies have shown that the number of initiation of dialysis was largest in the winter^12,13^. However, the sample size was relatively small, and these studies did not investigate peritoneal dialysis (PD) and kidney transplantation (KTx). The transition to all modalities of hemodialysis (HD), PD, and KTx was investigated in a study from the United States. However, as the conditions of the transition to RRT differ among countries^14^, it is unknown whether the same seasonal variations in the transition to RRT are observed in other countries.

Revealing seasonal variations in some diseases could have beneficial effects. First, the seasonality of the diseases may indicate that environmental factors are involved in the diseases. In some cases, interventions aimed at modifying the environmental factors may lead to reduced disease incidence and mortality. Second, healthcare resources may be allocated more effectively. Because the healthcare expenditure for patients with ESRD is increasing, better allocation of healthcare resources in patients with ESRD is necessary. Third, seasonal variations in various diseases may aid in interpreting the association between the diseases and other factors in clinical studies across different seasons.

In present study, we investigated seasonal variations in all-cause or cause-specific mortality in patients with ESRD and in the transition to RRT by using a large database from a Japanese nationwide registry for dialysis patients.

## Results

Table 1 shows the characteristics of patients in the analysis for the transition to RRT. Patients starting HD had the highest mean age, with the greatest proportion of patients ≥80 years old, whereas the patients receiving KTx had the lowest mean age, with the highest proportion of patients <60 years. The proportion of males was also lowest in the KTx patients. The proportion of patients with diabetes was highest in the HD patients and lowest in the KTx patients.

**Table 1:** Characteristics of the patients in the analysis of the seasonal variations in the transition to renal replacement therapy.

	Hemodialysis	Peritoneal dialysis	Kidney transplantation
	(N = 103,563)	(N = 5,902)	(N = 2,873)
Age (years)	68.6 ± 12.9	62.8 ± 14.1^a^	48.2 ± 13.1^a,b^
<60 (%)	21.3	37.8	74.9
60 =< <70 (%)	26.2	28.2	22.5
70 =< <80 (%)	31.1	21.6	2.5
80 =< (%)	21.4	12.2	0.1
Men (%)	67.5	67.8	63.0^a,b^
**Diabetes (%)**			
Diabetes	53.5	45.0^a^	22.0^a,b^
Non-diabetes	43.4	52	68.3
Unknown	3.2	3	9.7
**Difference of temperature (%)**			
Smallest	23.5	26.7^a^	27.5^a,b^
Second smallest	26.5	23.3	28.3
Second Largest	24.9	27.4	25.7
Largest	25.2	22.6	18.3
Others^#^	0	0	0.1

^#^Others mean patients who did not have data of living place or lived in a foreign country.

^a^P < 0.05 versus hemodialysis.

^b^P < 0.05 versus peritoneal dialysis.

**Figure 1:**
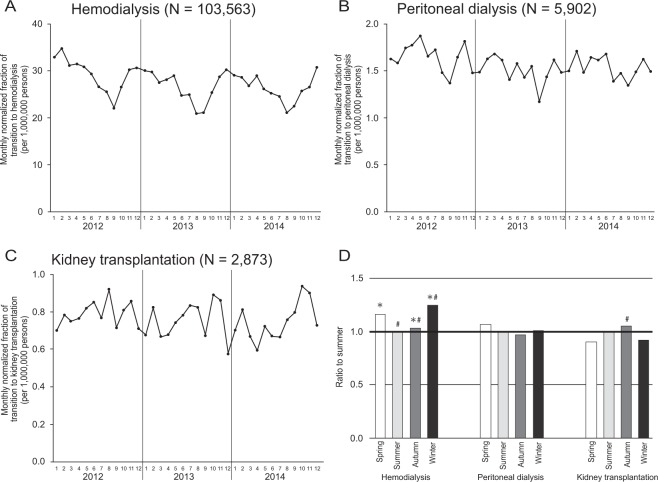
Monthly transition fraction to renal replacement therapy; (**A**) hemodialysis, (**B**) peritoneal dialysis, and (**C**) kidney transplantation. (**D**) Ratio of the transition fraction to each renal replacement therapy between the summer and the other seasons. *P < 0.05 versus summer. ^#^P < 0.05 versus spring.

**Figure 2:**
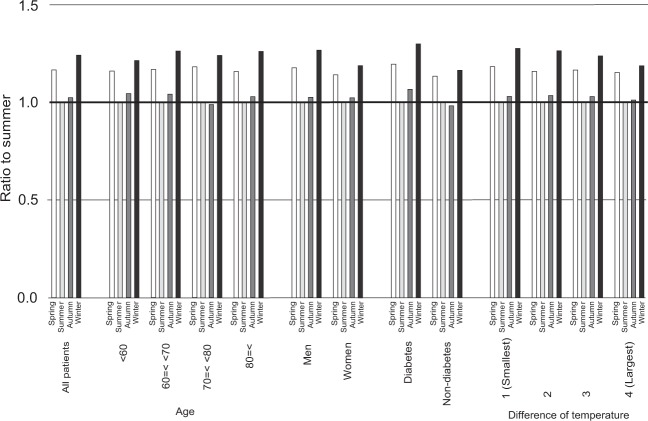
Ratio of the fraction of initiation of hemodialysis between the summer and the other seasons in subgroups.

Figure 1 shows the seasonal variations in the transition to RRT. The initiation of HD was highest in the winter and lowest in the summer. On the other hand, the initiation of PD was comparable among seasons. The transition to KTx was most frequent in the autumn and least frequent in the spring. The ratios of the highest to lowest average fraction of the transition to HD, PD, and KTx were 1.242, 1.099, and 1.161, respectively. In the subgroup analysis, the seasonal variations in initiation of HD were almost the same in all subgroups (Fig. 2).

Table 2 shows the characteristics of the patients in the analysis for mortality. HD patients had a higher mean age and had a higher proportion of patients >80 years of age, males, with diabetes, and with a past history of CVD than PD patients.

**Table 2:** Characteristics of the patients in the analysis of the seasonal variation in mortality.

	Hemodialysis	Peritoneal dialysis	P
	(N = 166,761)	(N = 5,774)	
Age (years)	67.1 ± 12.1	62.6 ± 13.1	<0.01
<60 (%)	23.5	37.4	
60 =< <70 (%)	31.3	32.6	
70 =< <80 (%)	29.8	19.8	
80 =< (%)	15.4	10.2	
Men (%)	63.7	61.9	<0.01
Diabetes (%)			<0.01
Diabetes	46.2	35.8	
Non-diabetes	53.8	60.8	
Unknown	0	3.5	
Past history of CVD (%)			<0.01
Past history of CVD	29.9	14.8	
No past history of CVD	70.1	32	
Unknown	0	53.2	
Difference of temperature (%)			<0.01
Smallest	26.7	23	
Second smallest	25.1	21.9	
Second largest	26.4	29.5	
Largest	21.8	24.6	
Others^#^	0	1.1	

^#^Others mean patients who did not have data of living place, lived in a foreign country, or moved to another place from 2012 to 2014.

CVD, cardiovascular disease.

**Figure 3:**
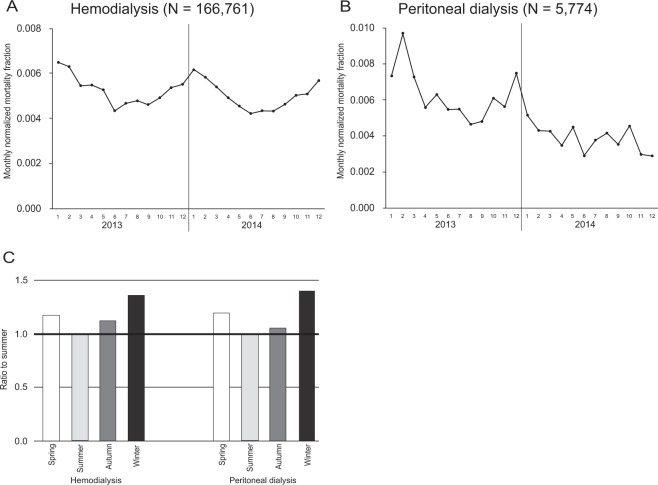
Monthly mortality fraction in (**A**) hemodialysis and **(B**) peritoneal dialysis. (**C**) The ratio of mortality between the summer and the other seasons in hemodialysis patients and peritoneal dialysis patients.

**Figure 4:**
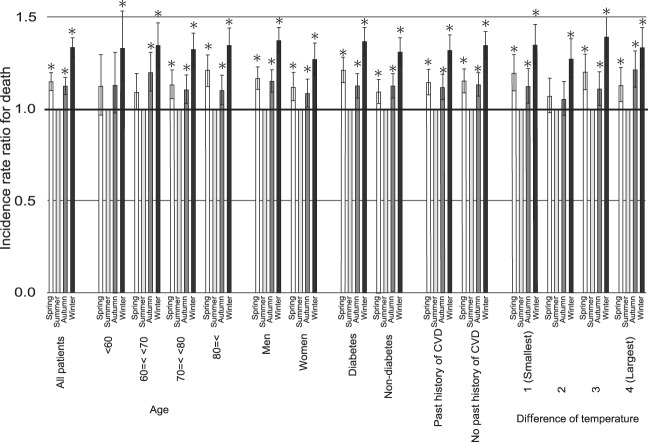
Unadjusted incident risk ratio for all-cause mortality in subgroups. *P < 0.05 versus summer.

All-cause mortality was highest in the winter and lowest in the summer in all groups (Fig. 3). A similar pattern was observed in all subgroups (Supplementary Table S1). In the mixed-effects Poisson analysis, the incidence rate ratio (IRR) for death in the winter was significantly highest among the seasons, and the IRRs in the spring and the autumn were significantly higher than that in the summer after adjusting for age, sex, diabetes, past history of CVD, and place of residence (Supplementary Table S2). Similar patterns were observed in each subgroup (Fig. 4).

**Figure 5:**
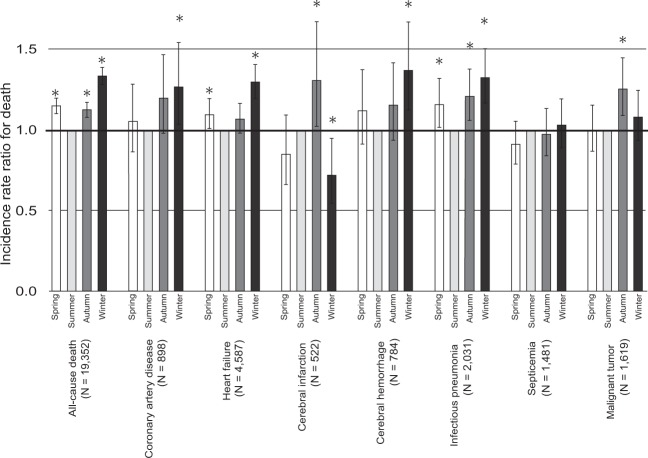
Unadjusted incident risk ratio for cause specific mortality. *P < 0.05 versus summer.

Figure 5 shows the results of seasonal variations in cause-specific mortality in HD patients. The IRRs for death from coronary artery disease, heart failure, cerebral hemorrhage, or infectious pneumonia in the winter were significantly higher than those in the summer. On the other hand, the IRRs of cerebral infarction, septicemia, or malignant tumor in the winter were not significantly higher than those in the summer.

## Discussion

This study showed that mortality in patients with ESRD and the initiation of HD were highest in the winter and lowest in the summer. These seasonal variations were observed in patients categorized by age, gender, diabetes, and place of residence. On the other hand, seasonal variations in the initiation of PD and transition to KTx were different from initiation of HD. Death rates from coronary heart disease, heart failure, cerebral hemorrhage, and infectious pneumonia in the winter were significantly higher than those in the summer, whereas death rates from cerebral infarction, septicemia, and malignant tumor did not show similar seasonality.

In regard to seasonal variations in mortality from CVD, heart disease and cerebral hemorrhage had a winter peak, whereas cerebral infarction did not show similar seasonality. Because, to the best of our knowledge, this is the first study to investigate the seasonal variations in heart disease and stroke in HD patients, it remains unclear whether this seasonality is confirmed in HD patients. However, this discrepancy between heart disease and ischemic stroke is observed in the general population^1^. A review reported that the incidence of heart failure and acute myocardial infarction increased in the winter, but in regard to stroke, 48.4% of the clinical studies showed a higher rate in the cold season, 39% of the studies showed it in the warm season, and 12% of the studies showed no seasonal variation^1^. It could also be noted that the amplitude was still small (IRR, 1.05 [1.04–1.07]), even though another meta-analysis showed a higher incidence rate of ischemic stroke in the winter than in the summer^4^. The reasons why seasonal variations in stroke are discordant are not completely clear. However, one of the reasons may be that cerebral infarction in our study included both cardioembolic stroke and atherothrombotic stroke, because one study in the general population has shown that the incidence of cardioembolic stroke was higher in the winter, whereas that of atherothrombotic stroke was higher in the summer^15^.

Several potential mechanisms, such as activation of the sympathetic nervous system, decrease in vitamin D, elevated coagulation factors, changes in hormones and vasoactive substances, and increase in respiratory infection, have been proposed to describe the higher winter mortality from heart disease in the general population^1,16,17^. In HD patients, elevated blood pressure was observed in the winter^8,9,18,19^. Additionally, because our study showed that the mortality from infectious pneumonia was highest in the winter, respiratory infection may partially contribute to the high mortality from heart disease in the winter. Intradialytic weight gain may be one of the causes of seasonal variation because it has been found to be largest in the winter^8,9,18,19^.

Our study has demonstrated that pneumonia infection had a winter peak, whereas septicemia did not have a seasonal variation in HD patients. Although previous studies have shown that the mortality from infection tends to be higher in the winter than in the summer in ESRD patients, these studies did not investigate each cause of infection^7,8^. A study in the general population has shown that respiratory infections had a large winter peak, genitourinary-related sepsis had a small summer peak, and sepsis originating from other sources of infection did not have a seasonal variation^2^. In another study, *Streptococcus pneumoniae* bacteremia had a large winter peak (peak-to-trough ratio, 3.42 [3.10–3.83]), *Escherichia coli* bacteremia had a small summer peak (1.17 [1.12–1.22]), and *Staphylococcus aureus* bacteremia had no seasonal variation (1.05 [1.00–1.13])^3^. Because *Staphylococcus aureus* bacteremia has been a major cause of death from bloodstream infection in HD patients^20^, those previous studies are likely to be in line with our results. In patients with ESRD, the incidence of influenza-like illness has been highest in the winter^10^, which supports our findings that mortality from pneumonia infection had a winter peak.

Death from malignant tumor has shown little or no seasonal variation^5,6,8^. A large clinical study including over 54 million deaths in 19 countries has shown that cancer mortality presented no substantial seasonality^5^. Another large clinical study including 959 307 malignant tumor deaths in Hungary between 1984 and 2013 has demonstrated that the mortality was highest in the winter. However, the amplitude was small (winter to summer ratio, 1.034)^6^. In HD patients, no significant seasonal variation has been shown^8^. Because our study also demonstrated no significant seasonal variation, malignant tumor may have little or no seasonality in HD patients.

The initiation of HD was highest in the winter and lowest in the summer, whereas seasonal variations in the initiation of PD and the transition to KTx were not similar to HD in our study. These results are consistent with previous studies^7,12,13^. Although the reason why the initiation of HD was highest in the winter remains unclear, acute kidney injury induced by heart failure, coronary artery disease, and infectious pneumonia may partially contribute to the seasonal variation in the initiation of HD. In fact, the incidence of acute kidney injury has been highest in the winter among hospitalized patients^21^. In addition, a multinational clinical study has demonstrated that most modality of RRT for acute kidney injury is HD^22^. Since incidence of acute kidney injury increase in winter and most modality of RRT for acute kidney injury is HD, initiation of HD may have winter peak, whereas since most of initiation of PD and transition to KTx are probably planned, they may have no or less seasonal variation.

Our study has several limitations. First, because the data in our study was collected by sending questionnaires, some of the data for causes of death may be misclassified. Second, because the data was collected from Japanese facilities, our results may not be applicable to other countries, especially those in a different climatic zone. However, because seasonal variations in the mortality of HD patients and the initiation of HD have been seen in other countries, seasonality may also be observed in different areas. Lastly, we did not adjust for factors other than age, sex, diabetes, past history of CVD, and place of residence. Specifically, laboratory data were not collected in each season and therefore could not be included. Despite these limitations, the strength of our study is the inclusion of a large number of patients that covers almost the entire population of dialysis patients in Japan. In addition, this is the first study investigating the seasonal variations in the specific causes of death in HD patients.

In conclusion, our study showed that the initiation of HD and the mortality from coronary heart disease, heart failure, cerebral hemorrhage, and infectious pneumonia in HD patients were more frequent in the winter and less frequent in the summer. On the other hand, similar seasonal variations were not observed in the initiation of PD and transition to KTx and the mortality of cerebral infarction, septicemia, and malignant tumor. Understanding the seasonal variations in the transition to RRT and mortality may provide some insight into the association between the disease and environmental factors, allow more efficient allocation of healthcare resources, and aid in the interpretation of clinical studies across seasons in ESRD patients.

## Methods

### Data source

We conducted the study using data from the database of the Japanese Society for Dialysis Therapy Renal Data Registry (JRDR). Details of this survey have been described elsewhere^23–25^. Briefly, the Japanese Society for Dialysis Therapy (JSDT) has been conducting annual surveys by sending questionnaires to all dialysis facilities in Japan at the end of each year. The response rate exceeded 98% every year, which means that the database of the JRBR covered almost all dialysis patients in Japan. The data set (JRDR-16009) in this study was created from the data for the years 2012, 2013, and 2014.

The estimated number of all people 20 years of age or older in Japan in each month from 2012 to 2014 was obtained from the Statistics Bureau, Ministry of Internal Affairs and Communications website (https://www.stat.go.jp/english/data/jinsui/2.html). The estimated number of all people 20 years of age or older in each prefecture was also obtained from the same website. However, because the data in each prefecture was collected annually, we used these same annual data in each prefecture as a substitute for the monthly population number in each prefecture.

The monthly mean temperature in each prefecture was substituted with the monthly mean temperature in the capital of each prefecture. The temperature in the prefectural capitals was obtained from the Japan Meteorological Agency website (https://www.data.jma.go.jp/obd/stats/etrn/index.php).

### Study population

Supplementary Fig. S1A shows the process for selecting study subjects in the analysis for the transition to RRT. Patients who initiated HD or PD were selected by excluding patients whose data for the day of first dialysis, death, KTx, or dialysis withdrawal, age, or dialysis modality were missing or incomplete. We excluded patients who started dialysis before 2011, who were <20 years of age at dialysis initiation, or whose dialysis modality was other than HD or PD. Patients who transitioned to KTx were defined as patients receiving a KTx from 2012 to 2014. In these patients, we excluded those whose data for age or date of receiving a KTx was missing or who were <20 years of age at the time they received a KTx.

Supplementary Fig. S1B shows the process for selecting study subjects in the analysis for mortality. Study subjects in this analysis were selected from patients with data at the end of 2012 because we performed subgroup analysis and mixed-effects Poisson regression analysis based on the data at the end of 2012. We also excluded patients whose data for the day of first dialysis, death, KTx, or dialysis withdrawal, age, or dialysis modality was missing or incomplete. Moreover, we excluded patients who were <20 years of age at the end of 2012, whose dialysis modality was other than HD or PD, or who received home dialysis. Study subjects in hemodialysis group were only patients who received HD three times weekly and whose HD time was from 9 to 18 hours per week because of the subgroup analysis and the multivariable mixed-effects Poisson regression analysis. Also excluded in these analyses were patients who did not have data for sex, diabetes, place of residence, or history of CVD or who lived in a foreign country or moved to another place from 2012 to 2014.

### Study design

We performed a descriptive analysis to evaluate seasonal variations in the transition to RRT and all-cause mortality. We also performed subgroup analysis for seasonal variations in the initiation of HD and all-cause mortality in HD patients. In addition, we performed a multivariable mixed-effects Poisson regression analysis for seasonal variation in mortality.

Subjects who have diabetic kidney disease or a past history of diabetes were defined as diabetic patients. Past history of myocardial infarction, cerebral infarction, cerebral hemorrhage, or amputation of the extremities was defined as a past history of CVD. Seasons were defined similarly with previous papers^8,9^. Winter was defined as December through February, spring as March through May, summer as June through August, and autumn as September through November. The temperature difference in each prefecture was calculated by subtracting the average of the mean monthly temperatures in the winter from the average of the mean monthly temperatures in the summer.

We divided the subjects into subgroup quartiles based on the difference in the average temperatures. The average temperature differences and prefectures in each category were as follows: smallest (17.76 °C ± 2.53 °C; Chiba, Kanagawa, Shizuoka, Kochi, Fukuoka, Nagasaki, Oita, Miyazaki, Kagoshima, and Okinawa), second smallest (20.25 °C ± 0.33 °C; Miyagi, Ibaraki, Tokyo, Mie, Hyogo, Wakayama, Shimane, Tokushima, Ehime, Saga, and Kumamoto), second largest (21.19 °C ± 0.20 °C, Tochigi, Saitama, Aichi, Shiga Osaka, Nara, Hiroshima, Yamaguchi, and Kagawa), and largest (22.54 °C ± 0.95 °C, Hokkaido, Aomori, Iwate, Akita, Yamagata, Fukushima, Gunma, Niigata, Toyama, Ishikawa, Fukui, Yamanashi, Nagano, Gifu, Kyoto, Tottori, Okayama).

The classification codes for the underlying cause of each death have been reported previously^26^. Coronary artery disease included the diseases coded as acute cardiac infarction and ischemic heart disease other than acute cardiac infarction. Heart failure included the diseases coded as cardiac failure and pulmonary edema. Cerebral hemorrhage included the diseases coded as subarachnoid hemorrhage and intracerebral hemorrhage.

The study protocol was approved by the Ethics Committee of the JSDT (approval number 14). The study was exempt from the need to obtain informed consent from participants, which was also approved by the Ethics Committee of the JSDT. The study was conducted in accordance with Helsinki Decleration.

### Statistical analysis

We described the clinical characteristics using mean and standard deviation for continuous variables or percentage for categorical variables. A comparison of continuous variables was performed using unpaired t-test or using one-way ANOVA with *post hoc* Bonferroni correction. A comparison of proportion between groups was performed using chi-square test and Bonferroni correction was applied when multiple comparison was needed.

The number of outcomes per month was normalized based on the method reported by Obi *et al*.^7^. With this, the equation is as follows: (monthly normalized number of outcomes) = (monthly number of outcomes)/(days in a given month) × ([365 × 3] + 366)/4/12. Monthly normalized fractions of the transition to RRT were calculated by dividing the normalized number of the transition to RRT per month by the number of all people 20 years of age or older in Japan at the beginning of the month. Monthly normalized fractions of the transition to RRT in the subgroup analysis were calculated by dividing the normalized number of the transition to RRT per month by the number of all people in the subgroup in Japan. For example, the fractions of the transition to RRT per month in the subgroup of males were calculated by dividing by the number of men 20 years of age or older in Japan at the beginning of the month. However, the fractions in the subgroups of diabetes and non-diabetes were calculated by dividing by the number of people 20 years of age or older in Japan. This was done because we could not find the number of all diabetic or non-diabetic patients 20 years of age or older in Japan. Moreover, all-cause and cause-specific monthly normalized mortality fractions were calculated by dividing the normalized number of deaths per month by the number of HD patients who were alive at the beginning of the month. The ratio of winter to summer was calculated by dividing the average of the monthly normalized fraction in January, February, and December by the average of the monthly normalized fraction in June, July, and August.

We also performed mixed-effects Poisson regression analysis to compare mortality among seasons and IRR for all-cause mortality in HD patients was adjusted for some variables. First, we expanded the data for each patient to data for each patient per seasons. For example, patients who survived until the end of the study period had data for nine time points (first winter, first spring, first summer, first autumn, second winter, second spring, second summer, second autumn, and third winter) because our study period in the analysis for mortality was from January 2013 to December 2014. We used this panel of data for the mixed-effects Poisson regression analysis. In the mixed-effects Poisson regression analysis, we set each patient as a random effect; seasons, age, sex, diabetes, past history of CVD, and place of residence as fixed effects; and days of each season as an offset variable. If a patient died or dropped out, days of the last season for the patient were calculated as follows: days of the last season for the patient = (days in the month before the death or dropout during the season) + (days in month of the death or dropout)/2. For example, if a patient died in May, the days of the last season for the patient were 76.5 days (31 + 30 + 15.5).

*P* value < 0.05 was considered to be statistically significant. All statistical analyses were performed using Stata/MP 14.2 software for Windows (Stata, College Station, TX, USA).

## Data availability

The data that support the findings of this study are available from JSDT but restrictions apply to the availability of these data, which were used under license for the current study, and so are not publicly available. Data are however available from authors upon reasonable request and with permission of JSDT.

## Supplementary information

Supplementary Material.
