# Supplementary material for: Seasonal variations in cause-specific mortality and transition to renal replacement therapy among patients with end-stage renal disease
Source: Sci Rep. 2020 Feb 11;10:2325. doi: 10.1038/s41598-020-59153-6 (PMC7012814; doi:10.1038/s41598-020-59153-6)
Supplement: Supplementary file 1 — Supplementary Material. [file 41598_2020_59153_MOESM1_ESM.pdf]

Supplementary Material

Seasonal variations in cause-specific mortality and transition to renal replacement therapy among patients with end-stage renal disease

Shunsuke Goto[1, 2], Takayuki Hamano[1, 3], Satoshi Ogata[1, 4], and Ikuto Masakane[1, 5]

1. Committee of Renal Data Registry, Japanese Society for Dialysis Therapy, Tokyo, Japan

2. Division of Nephrology and Kidney Center, Kobe University Graduate School of Medicine, Kobe, Japan

3. Department of Inter-Organ Communication Research in Kidney Disease, Osaka University Graduate School of Medicine, Suita, Japan

4. Department of Clinical Nutrition, Hiroshima International University, Kure, Japan

5. Department of Nephrology, Honcho Yabuki Clinic, Yamagata, Japan

Supplementary Table S1. Mortality ratio between the summer and the other seasons in subgroups of hemodialysis patients

	spring	summer	autumn	winter
All patients	1.165	1.000	1.112	1.350
Age (years)				
<60	1.124	1.000	1.128	1.336
60=< <70	1.092	1.000	1.196	1.347
70=< <80	1.140	1.000	1.096	1.330
80=<	1.224	1.000	1.092	1.354
Sex				
Men	1.182	1.000	1.136	1.389
Women	1.134	1.000	1.070	1.282
Diabetes				
Diabetes	1.209	1.000	1.126	1.363
Non-diabetes	1.119	1.000	1.099	1.336
Past history of CVD				
Past history of CVD	1.174	1.000	1.094	1.348
No past history of CVD	1.151	1.000	1.133	1.346
Difference of temperature				
Smallest	1.214	1.000	1.103	1.367
Second smallest	1.079	1.000	1.046	1.280
Second largest	1.220	1.000	1.092	1.408
Largest	1.140	1.000	1.203	1.339

CVD, cardiovascular disease.

Supplementary Table S2. Adjusted incidence rate ratio for death associated with seasons

	Model 1 (Unadjusted)	Model 2	Model 3	Model 4
Spring	1.15 [1.10–1.20]	1.16 [1.11–1.20]	1.15 [1.10–1.20]	1.15 [1.10–1.20]
Summer [Ref.]	1.00	1.00	1.00	1.00
Autumn	1.13 [1.08–1.17]	1.12 [1.07–1.16]	1.12 [1.08–1.17]	1.12 [1.08–1.17]
Winter	1.34 [1.28–1.39]	1.34 [1.29–1.40]	1.33 [1.28–1.39]	1.33 [1.28–1.39]
Men		1.22 [1.18–1.26]	1.14 [1.11–1.18]	1.14 [1.10–1.18]
Age (year)		1.07 [1.07–1.07]	1.07 [1.07–1.07]	1.07 [1.07–1.07]
Diabetes			1.21 [1.17–1.25]	1.21 [1.18–1.25]
Past history of CVD			1.93 [1.86–2.00]	1.93 [1.86–2.00]

CVD, cardiovascular disease.

Model 2: adjusted for sex and age. Model 3: adjusted for covariates in Model 2, diabetes, and past history of CVD. Model 4: adjusted for covariates in Model 3 and places of residence.

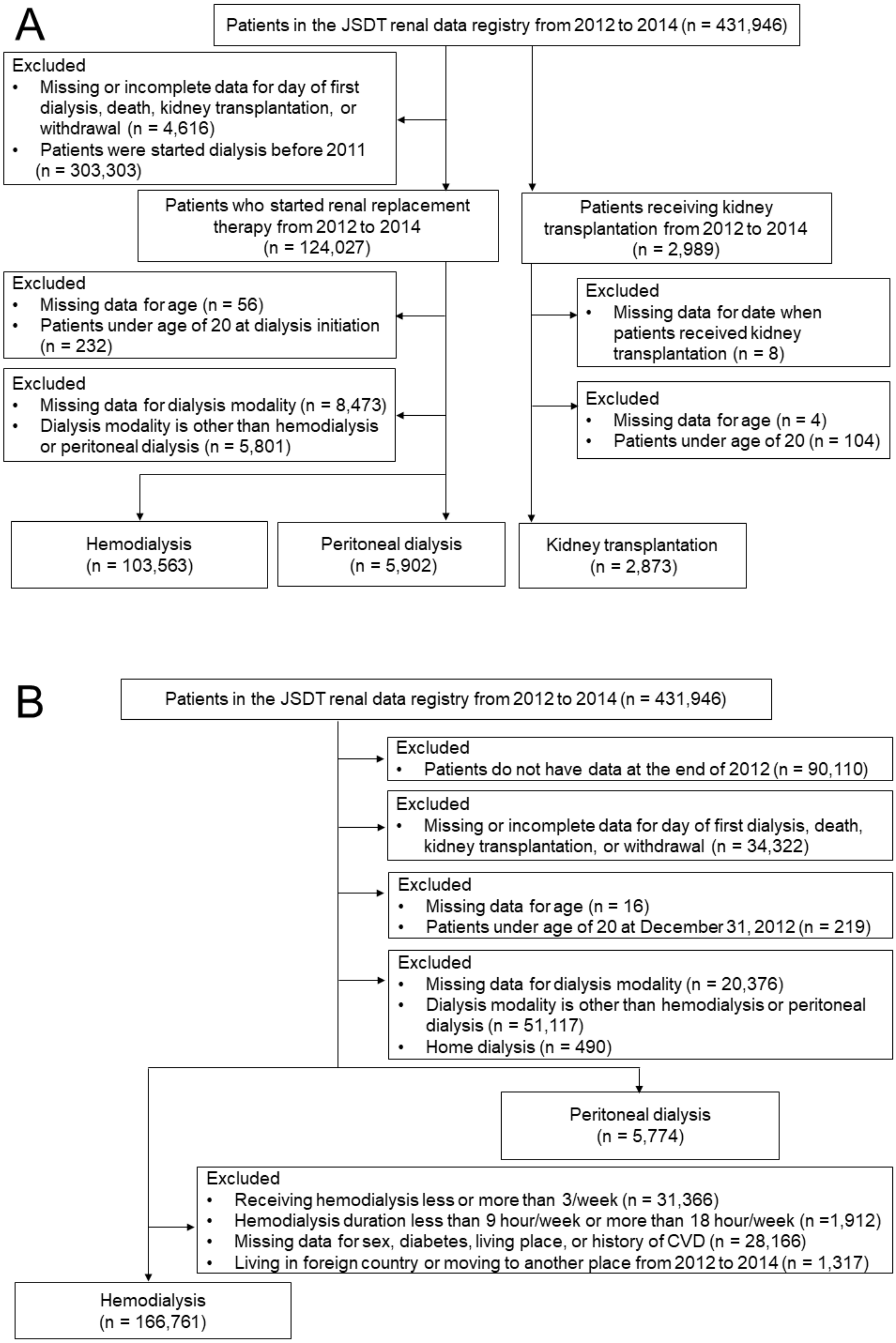

Supplementary Figure S1. Flow diagram in (A) the analysis of seasonal variation in the transition to renal replacement therapy and (B) the analysis of seasonal variation in mortality.
